# Cardiac Magnetic Resonance for Evaluating Catheter Related FDG Avidity

**DOI:** 10.1155/2016/5460727

**Published:** 2016-10-27

**Authors:** Daniel Jeong, Kenneth L. Gage, Claudia G. Berman, Jaime L. Montilla-Soler

**Affiliations:** Department of Radiology, H. Lee Moffitt Cancer Center, Tampa, FL 33612, USA

## Abstract

A 53-year-old female with a history of metastatic left arm melanoma presented for F(18) fluorodeoxyglucose (FDG) positron emission tomography/computed tomography (PET/CT) which showed a moderately FDG avid focus at her port catheter tip near the cavoatrial junction. Although catheter tip related FDG avidity has previously been suggested to be bland thrombus or infection, melanoma can metastasize to unusual locations including the superior vena cava. In addition, the patient had an elevated risk of anticoagulation due to a history of hemorrhagic brain metastases. Therefore, confirmatory cardiac magnetic resonance (CMR) was obtained and findings were consistent with bland catheter-related thrombus.

## 1. Introduction

Cancer patients who receive PET/CT exams often have indwelling central catheters and ports. Rare reports have suggested catheter related FDG activity related to bland thrombus or infection [1, 2]. Bhargava et al. reported a series where 3 out of 4 patients with elevated FDG activity at the distal end of a central catheter were related to bland thrombus and 1 had clinical infection [2]. Each of the 4 FDG injections in this series was through a peripheral vein separate from the catheter [2]. Siösteen et al. presented a case of FDG activity at a catheter tip related to bland thrombus with the FDG injection occurring through the same catheter due to poor vascular access [1]. When FDG activity occurs at the superficial injection site of the port or port cavity, this is typically related to infection [3]. In addition, confirmatory diagnostic imaging of catheter tip related FDG avidity has not been previously presented in the literature.

## 2. Case Presentation

**Figure 1:**
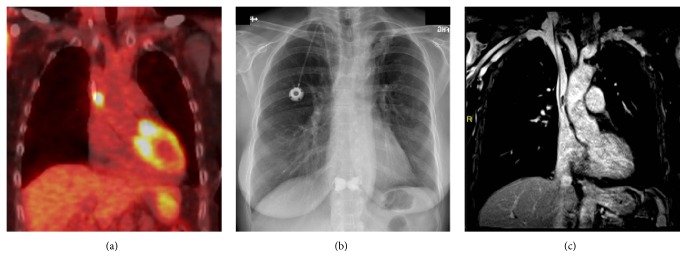
PET/CT, radiograph, and MR correlation. (a) Coronal reformatted PET/CT fusion image shows a moderately hypermetabolic focus at the approximate location of the catheter tip. SUV max was 8.8. (b) PA chest radiograph demonstrates a right chest wall port with its catheter tip at the cavoatrial junction. (c) Coronal T1 MR venous phase postcontrast image demonstrates a linear hypoenhancing structure along the medial lower SVC wall abutting the distal catheter. This finding was compatible with catheter related bland thrombus.

**Figure 2:**
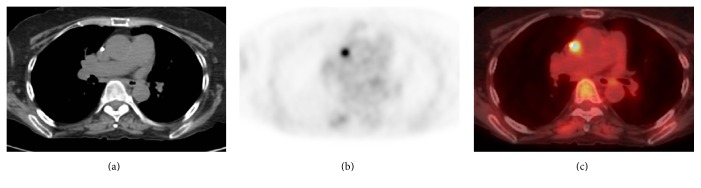
FDG PET/CT exam. Axial CT (a), PET (b), and (c) fused images from PET/CT exam demonstrate moderately hypermetabolic focus correlating with the catheter tip on CT.

**Figure 3:**
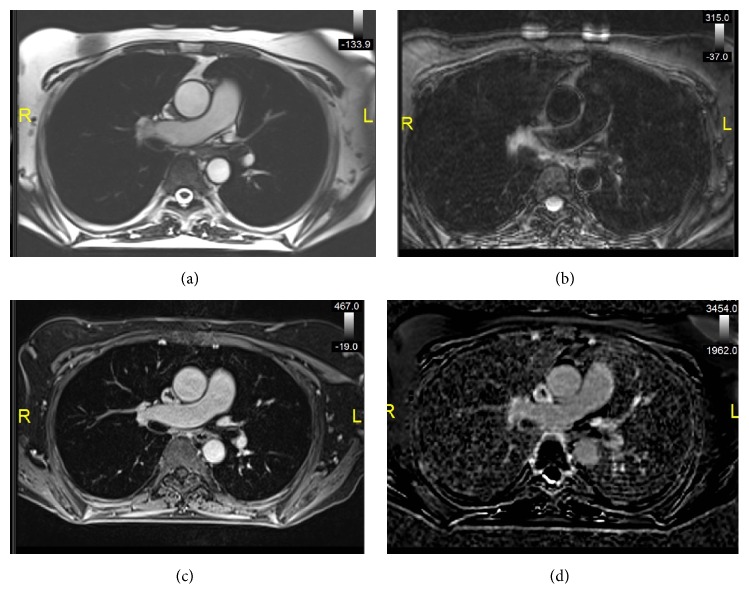
Cardiac magnetic resonance evaluation. (a) Axial SSFP image shows a hypointense structure conforming to the medial lower SVC wall. (b) Axial T2 SPAIR MR image shows isointense T2 signal within the structure. (c) Axial T1 postcontrast image demonstrates a well-defined intravascular filling defect along the SVC wall. (d) Late gadolinium enhancement phase sensitive inversion recovery image demonstrates hypoenhancement of the intravascular structure. Findings are compatible with bland thrombus.

A 53-year-old female with a remote history of melanoma of the left arm had recurrence to the chest wall and brain. She presented for routine restaging positron emission tomography/computed tomography (PET/CT) (Figures 1(a) and 2(a)–2(c)) which showed an F(18) fluorodeoxyglucose (FDG) avid focus in the lower superior vena cava (SVC) just above the cavoatrial junction adjacent to the catheter tip of her right chest wall port. Maximum standard uptake value (SUV max) was 8.8. Due to poor vascular access, the patient's port was utilized to administer the FDG dose of 11.9 mCi for the exam. The patient had no clinical signs of catheter related infection. Figures 1(a) and 1(b) show the FDG avid focus on coronal fused PET/CT and the port catheter on frontal chest radiograph, respectively. Although catheter-related thrombus was initially suspected, the patient had a recent history of hemorrhagic brain metastases with an increased risk for anticoagulation. In addition, the SUV max of 8.8 was relatively higher than previous reports of bland thrombus. Cardiac magnetic resonance (CMR) was recommended for imaging confirmation. CMR demonstrated a hypointense lesion along the medial superior vena cava wall just above the catheter tip. The lesion demonstrated hypointense T1- and iso- to hypointense T2-weighted signal (Figures 1(c) and 3(a)–3(d)). No appreciable enhancement was noted on postcontrast (gadobutrol, 0.15 mmol/kg) or delayed gadolinium images. CMR findings were consistent with bland catheter-related thrombus.

## 3. Discussion

In higher risk cases such as ours, it can be clinically relevant to fully exclude tumor thrombus prior to initiating anticoagulation therapy. Ghattas et al. reported a case of melanoma of the left axilla with intravascular metastasis to the lower SVC which was also hypermetabolic on PET/CT, but with no central catheter present [4]. Bland non-catheter-related thrombus often has a lower SUV max compared to tumor thrombus. In general, SUV max greater than 3.35–3.63, contiguous intravascular spread from larger tumor, and location in the IVC have all been associated with tumor rather than bland thrombus [5–7].

Some authors suggest that an inflammatory process surrounding chronic bland thrombus is responsible for associated FDG avidity [1, 2, 5]. When FDG activity occurs at the tip of the catheter that was used for the injection, physical FDG trapping becomes another possibility [1]. Patients with long term indwelling central catheters can develop sleeve thrombus or an associated fibrin sheath that could theoretically cause physical trapping of FDG [1, 8]. In our case, the central port catheter with FDG activity finding at the tip was utilized for the FDG injection due to otherwise poor vascular access. Therefore physical bland thrombus/sheath related FDG trapping or associated inflammation are plausible explanations for FDG activity in our case.

CMR provides a unique diagnostic tool for evaluating a hypermetabolic cavoatrial junction mass. Cine balanced steady state free precession (BSSFP) images allow cardiac gated evaluation of thrombus which presents as a hypointense structure surrounded by normal bright blood signal. Acute thrombus can present with intermediate to hyperintense T1- and T2-weighted signal [9, 10]. Subacute thrombus appears hyperintense on T1-weighted images with hypointensity on T2-weighted images [9]. Chronic thrombus appears hypointense on T1- and T2-weighted images [9]. Thrombus presents as a filling defect on postcontrast sequences compatible with its avascular nature.

While lesions in the lower SVC may be imaged on postcontrast MR angiogram, T1-weighted sequences, or even CT angiography (CTA) without cardiac gating, lesions within the atrial cavity are subject to artifact from cardiac motion. Therefore cine and cardiac gated images provide a significant advantage in evaluating catheter related lesions which can often be located within the right atrium. Compared to CTA, real time gated perfusion imaging in CMR allows better detection of subtle enhancement with added benefit of temporal resolution of the enhancement. In addition, T2-weighted and late gadolinium enhanced imaging further increase specificity in mass evaluation compared to CTA.

Melanoma metastases demonstrate intrinsically hyperintense T1-weighted signal similar to that expected with subacute thrombus. Therefore, postcontrast imaging becomes a more important sequence to distinguish bland versus melanoma tumor thrombus. In our case, low T1- and T2-weighted signal without postcontrast enhancement were compatible with chronic bland thrombus. While Rinuncini et al. used CMR to diagnose a hypometabolic left ventricle mass as chronic bland thrombus, our case is the first to apply CMR to confirm bland thrombus at the cavoatrial junction associated with catheter tip FDG avidity [11]. Additionally, the emergence of PET/MR scanners will offer more clinical avenues for which PET can be obtained in similar cases. In this case, PET/MR would have provided seamless integration of the MR data needed to confidently diagnose the FDG avid focus as bland thrombus.

In conclusion, CMR is an excellent tool to further evaluate intravascular FDG avidity in the SVC, heart, and other major surrounding vessels. FDG avidity at the tip of a central catheter has classically been reported as bland. However, in cases where anticoagulation therapy has elevated risks and for primary tumors known to have unusual metastases such as melanoma, CMR should be the recommended next diagnostic exam.
